# Bridging Over Troubled Waters—How the United States 2018 Heart Allocation System Altered Transplant Bridging Strategies

**DOI:** 10.31083/j.rcm2502068

**Published:** 2024-02-20

**Authors:** Les James, Deane E. Smith

**Affiliations:** ^1^Department of Cardiothoracic Surgery, NYU Langone Health, New York, NY 10010, USA

**Keywords:** heart transplantation, bridging strategies, mechanical support

## Abstract

As we approach the five-year anniversary of the 2018 heart allocation system in the United States, it is imperative to consider the changing landscape of mechanical circulatory support and the strategies used to bridge patients into heart transplants. This manuscript reviews the history of the heart allocation system, as well as the conditions that led to its multiple revisions. We discuss initial outcomes following the implementation of the new allocation system, including the impact on waitlist mortality and post-transplant outcomes. We also give special consideration to changes in bridging strategies using venoarterial extracorporeal membrane oxygenation (VA ECMO), intra-aortic balloon pumps, and durable left ventricular assist devices (LVADs).

## 1. Introduction

Heart transplantation is the gold standard of treatment for patients with end-stage heart failure. Over the past decade, the number of total heart transplants (HT) performed annually in the United States (U.S.) has grown each year; in 2022 there were over 4000 HT—an all-time record [1]. Despite the increasing number of HT performed year after year, the organ donor pool has failed to keep pace; from 2008 to 2019, new listings for HT increased by 42.5% [2, 3]. As a consequence of the discrepancy between donor supply and organ demand, mechanical circulatory support (MCS) has emerged to provide either temporizing or definitive solutions for patients with end-stage heart failure.

Temporary MCS can be utilized for a number of indications, including high-risk percutaneous coronary interventions, cardiogenic shock (CS) refractory to medical therapy, myocardial recovery, and as a bridge to definitive therapy, either durable MCS or HT. Currently available temporary MCS devices include intra-aortic balloon pumps (IABPs), percutaneous ventricular assist devices (pVADs) such as Impella, venoarterial extracorporeal membrane oxygenation (VA ECMO), and surgically placed temporary ventricular assist devices (VADs). Durable MCS devices, most commonly left ventricular assist devices (LVADs), are designed for long-term support and implanted in patients with advanced heart failure as either a bridge to transplantation (BTT) or destination therapy (DT) strategy. Patients requiring biventricular support have poor rates of survival to HT, and as such biventricular assist devices (BiVADs) are only utilized in approximately 5% of patients with MCS devices [4, 5]. For patients not yet listed for HT or for whom eligibility has not yet been determined, implantation of a durable MCS device can be performed as a strategy of “bridge to candidacy” (BTC). For patients with the highest degree of uncertainty, implantation of a durable MCS device is sometimes categorized as a “bridge to decision”.

In October 2018, Organ Procurement and Transplantation Network/United Network for Organ Sharing (OPTN/UNOS) modified the heart allocation system and transitioned from a three-tiered to a six-tiered system. The strategic aim of the allocation change was to “provide equity in access to transplants” by increasing rates of transplantation for candidates at the highest risk for waitlist mortality. In this vein, the new allocation system aimed to more accurately distinguish listed patients with varying levels of medical urgency, minimize waitlist time, and improve geographic disparities in organ availability. An unintended and much discussed consequence of the new allocation system has been a considerable shift in the strategies used to bridge patients to either transplant, candidacy, or decision.

As we approach the five-year anniversary of implementing the 2018 heart allocation system, a critical question is how the bridging landscape has evolved and what the impact has been on waitlist mortality and post-transplant outcomes. This review article will discuss a brief history of the U.S. heart allocation system, as well as the conditions that led to its revision. Initial outcomes following the implementation of the new allocation system will be highlighted, with special consideration given to changes in bridging strategies using VA ECMO, IABP, Impella, and durable LVADs.

## 2. A History of the Heart Allocation System

In 1968, the U.S. Congress passed the Uniform Anatomical Gift Act, which established the regulatory framework for adults to register as organ donors. The National Organ Transplant Act of 1984 subsequently created the OPTN, a system to keep track of individuals needing organ transplants and facilitate matching organs with appropriate patients [6]. In 1986, the Health Resources and Services Administration of the Department of Health and Human Services (DHHS) contracted with the UNOS to maintain the OPTN and ensure the fair and equitable allocation of transplants. Additionally, UNOS works with hospitals and organ procurement organizations to match transplant candidates with donated organs through organ allocation. Allocation is based upon the combination of urgency (i.e., candidates with the highest pretransplant mortality) and potential benefit gained from transplantation (i.e., those with the highest post-transplant survival) [7]. Four years after the introduction of the National Organ Transplant Act, OPTN/UNOS finalized the first heart allocation system. Heart allocation was initially a point-based algorithm largely based on the renal allograft distribution model. This first heart allocation model was in effect between 1989 and 1999 and functioned as a two-tiered heart allocation system based upon urgency, waiting time, geography, and blood type [8, 9]. Criteria for Status 1 included the need for intensive care unit (ICU)-level care with the use of inotropes, continuous intubation, or any MCS, while all other listed candidates were Status 2. Since its inception, there have been two major revisions to the heart allocation system: the creation of a three-tiered system in 1999 and prioritization of urgency over regional borders in 2005.

For several decades, HT was the only life-prolonging treatment for patients with end-stage heart failure. As patients in need of transplantation began to outpace the relatively static donor pool, there was a steady increase in waitlist mortality [10]. Longer waitlist times also resulted in patients clinically deteriorating to the point of no longer being suitable transplant recipients [11]. The combination of a limited number of available organs and increasing waitlist mortality spurred the development of MCS devices that would allow patients to survive in the outpatient setting until a donor heart became available. In 1994, the U.S. Food and Drug Administration approved the first pneumatically driven LVAD as a BTT, introducing the possibility of stabilizing patients to await eventual transplantation without mandating hospitalization [12]. In light of evolving MCS therapies and with broad input from the transplant community, in 1999 the original two-tiered system was revised to refine the details of priority status listing. The primary goal of this revision was to direct hearts to patients with the highest medical urgency and reduce both time and mortality while awaiting transplantation. The result was a three-tiered system: Status 1A (high priority), Status 1B (intermediate priority), and Status 2 (low priority). It is worth noting that patients implanted with the first generation of continuous flow LVADs suffered from excessive early mortality, prompting the OPTN/UNOS to allow listing these patients at the highest urgency status (Status 1A) within the first 30 days of LVAD implant [13]. This grace period was intended to mitigate the risk of post-LVAD implant complications and maximize the benefit obtained from HT by allowing an interval for recovery after receiving a durable device. As postoperative care for patients with newly implanted LVADs improved, in 2002 the heart allocation system was again modified to allow the 30-day highest urgency status to be used at the discretion of the clinical team [6, 14]. Importantly, use of this status exception did not require the patient to be hospitalized.

The Final Rule, originally proposed by the DHHS in 1998 and implemented in 2000, mandates that organ allocation should not be based on the transplant candidate’s place of residence or listing to avoid wasting organs and to promote access and efficiency [15]. In 2005, to reduce waitlist mortality for the most critically ill patients, OPTN/UNOS expanded regional organ sharing to facilitate organ distribution to the sickest candidates over larger geographic areas. This change initially produced the desired effect of increasing the availability of hearts for Status 1A and 1B candidates, statuses which encompassed all LVAD patients. The 1999 OPTN/UNOS annual report demonstrated that patients transplanted as Status 1A, 1B, and 2 accounted for 34%, 36%, and 26% of transplants, respectively, by comparison, in 2008 the proportions for Status 1A, 1B, and 2 were 54%, 37%, and 9%, respectively [16]. Although mortality on the waitlist initially declined [17], between 2006 and 2015 the number of active HT candidates nearly tripled (1203 vs. 3008 candidates) and the number of Status 1A candidates increased five-fold. In 2014, more than half of adult HT were performed in Status 1A patients, and waitlist mortality within this echelon varied widely—from 4.8% in candidates with an LVAD infection to 35.7% in candidates supported with VA ECMO [18].

Continued advances and refinements in LVAD technology, including miniaturization of pumps and improved patient selection, revolutionized MCS and morbidity and mortality associated with LVADs steadily declined. The Multicenter Study of MagLev Technology in Patients Undergoing Mechanical Circulatory Support Therapy with the HeartMate3 (MOMENTUM3) trial compared the HeartMate 3 centrifugal-flow LVAD to the HeartMate II axial-flow device in patients with advanced-stage heart failure, and found that the centrifugal-flow LVAD was associated with less frequent need for pump replacement and was superior with respect to survival free of stroke or reoperation to replace or explant a malfunctioning device [19]. In a review of over 15,000 patients collected by the Scientific Registry of Transplant Recipients (SRTR) between 2005 and 2010, Dardas *et al*. [18] demonstrated that stable LVAD patients have a low risk of adverse events, defined as death prior to HT or waitlist removal for clinical deterioration (1% cumulative hazard), when compared to Status 1A patients on dual inotropes (6% cumulative hazard) and Status 1A patients with pVAD (15% cumulative hazard) [20]. Long term survival of patients with LVADs also improved, especially when comparing the 2008–2012 era with more recent years [4]. Since 2013 LVAD survival outcomes have remained relatively unchanged (12-months: 82%; 24-months: 72%) [21]. Simultaneously, outcomes for patients BTT with LVADs were increasingly more successful. LVAD prior to transplantation, for any duration of time, resulted in no differences in long term post-transplant outcomes compared to patients undergoing *de novo* HT [22, 23]. By 2018, nearly one-half of HT recipients were on LVAD support at the time of transplantation [10, 24].

Thus, there were multiple limitations within the three-tiered heart allocation system. While transplant centers proliferated, and the criteria for HT expanded to include older, obese patients with more comorbid conditions and a greater prevalence of smoking and medication nonadherence, the donor pool remained relatively stagnant [7]. Women, Hispanics, and patients with restrictive/dilated cardiomyopathies or congenital heart disease were more likely to die while awaiting HT compared to men, white patients, and those with either ischemic or dilated cardiomyopathy. Hsich succinctly summarized these issues as underlying the need for improved “matching of the market” by increasing the donor pool, reducing the waitlist, and improving the allocation system [25]. As a result of these emerging data and concerns that the existing system was inequitable, the OPTN/UNOS Heart Subcommittee initiated the process of refining the allocation criteria, leading to a newly revised allocation system in December 2016 that was implemented in October 2018 [26].

## 3. 2018 OPTN/UNOS Revision to Heart Allocation System

On October 18, 2018 OPTN/UNOS implemented a revised heart allocation system that intended to address inequities and deficiencies of the previous system. These included an overabundance of candidates with highly diverse urgency within the Status 1A classification, increasing the dependence on exception requests, the increased utilization of MCS and its attendant complications unaccounted for in the policy, and a geographic sharing scheme that was inconsistent with the Final Rule [27]. The primary strategic goal of the new policy was to ensure equitable access to transplants by increasing rates of transplantation for candidates at high risk for waitlist mortality. The new six-tiered system was significantly more complex. It aimed to improve discrimination among listed candidates with distinct levels of urgency for the most critically ill patients, account for contemporary uses of MCS, expand standard criteria to include the most common reasons for exception requests, and promote broader sharing of donor hearts. Standardized definitions for CS, refractory ventricular arrhythmias, and durable VAD complications were enacted to reduce the impact of variability in clinical practice on listing practices across regions and transplant programs. Lastly, a broader distribution strategy was developed to expand access for Status 1 and 2 candidates and to guard against regional “isolationism”, and the regional review board process was modified such that exception requests were no longer reviewed in the same region from which they were submitted [27].

With respect to MCS and bridging strategies, expansion from a three-tiered to six-tiered allocation system aimed to improve risk stratification for the heterogeneous group of patients previously listed as Status 1A by combining candidates with similar waitlist- and post-transplant mortality. Under the prior system, patients with durable LVADs were eligible to be listed as Status 1A for 30 days or if they were experiencing a life-threatening device complication; otherwise, they were listed as Status 1B. The revised allocation system enacted uniform definitions for device-related complications and patients supported with durable LVADs were largely redistributed to lower tiers, reflecting their improved waitlist survival over the last decade. Under this system, stable LVAD patients are Status 4, with increased urgency for those with life-threatening ventricular arrhythmias (Status 1), non-dischargeable patients or those with device failure (Status 2), or those with other device-related complications (e.g., device infection, hemolysis, pump thrombosis, aortic insufficiency, and right heart failure) (Status 3). Patients still have access to a 30-day discretionary period at a higher priority listing (Status 3). The new allocation system also requires specific physiological criteria to meet some tier indications, such as the definition of CS (cardiac index <2.0 L/min/$m^{2}$ with a systolic blood pressure <90 mmHg and a pulmonary capillary wedge pressure >15 mmHg). Additionally, for candidates at lower priority statuses, transplant centers must now justify the need for high-dose inotropes. While beyond the scope of changes in bridging strategies, other aspects of the revised allocation system included a mandate for organ sharing over larger areas without regard to governmental boundaries and explicit tier assignments for patients with restrictive/dilated cardiomyopathies or congenital heart disease (Status 4).

In summation, these changes comprehensively restructured the heart allocation system. For many, the primary measure of success was whether the 2018 allocation system could optimize pre- and post-transplant morbidity and mortality and simultaneously curb excessive waitlist times. Urgency continues to be based on the need for MCS or ventilatory support, although there are no standard criteria used to initiate these interventions. An alternative approach would be the use of a heart allocation score, similar to the algorithmic models used to predict mortality for liver and lung transplantation (e.g., Model for End-Stage liver Disease [MELD], and the Lung Allocation Score [LAS]) [7]. While the possibility of a heart allocation score was debated by the OPTN/UNOS Heart Subcommittee, it was ultimately abandoned given the time projected to develop such an algorithm and the anticipated complexities of modeling rapidly evolving technology for the support of patients with end-stage heart failure [9]. Nevertheless, early evidence suggests that the system achieved many of its intended goals, including improved stratification of candidates by medical urgency and broader distribution of organs with minimal impact on overall post-transplant outcomes [27]. However, there were unintended consequences for bridging strategies. Patients in CS could be treated with inotropes, temporary MCS, or durable MCS, all at the discretion of the treating physician, but with vastly different implications for listing status and probability of eventual progression to transplantation.

## 4. New Strategies for Bridging to Heart Transplant

### 4.1 Initial Trends and Outcomes 

In one of the first reports of outcomes following the implementation of the new heart allocation system, Cogswell and colleagues [28] analyzed data from the publicly available UNOS registry. Patients listed and transplanted in the three years before the UNOS allocation system (n = 6001; October 18, 2015–October 18, 2018) were compared with those listed and transplanted under the new system (n = 539; October 18, 2018–March 31, 2019). Those listed and transplanted in the new system were more likely to (1) require temporary MCS and (2) have worse hemodynamics (i.e., higher pulmonary vascular resistance, lower cardiac output, and higher mean pulmonary capillary wedge pressures). Additionally, in the new system, 83% of transplants occurred in urgency Status 1, 2, or 3 [28]. As patients were being listed for HT at higher statuses, time on the waitlist was shorter and waitlist mortality decreased. It is important to note that decreased waitlist time was not solely a function of the new allocation change; the use of extended-criteria donors (e.g., hepatitis C positive cardiac allografts), deceased cardiac donors, and increased overdose death donors all were likely contributors to decreased waitlist time. At the same time, estimated 90-day survival was noted to be significantly lower under the new system compared to the prior system (87.6% vs. 94.5%, *p* < 0.0001), as was 180-day survival (77.9% vs. 93.4%, *p* < 0.0001). In multivariate models, patients listed and transplanted in the new system also experienced a higher hazard rate for death or re-transplantation [28]. While some of the studies that followed also demonstrated increased post-transplant mortality under the new allocation system [29, 30], others did not despite using the same UNOS database [27, 31]. These inconsistencies were attributed to differences in study population, time periods, and follow-up time included within the analysis. More recently, a report published in October 2022 entitled, “Three-Year Monitoring of Heart Allocation Proposal to Modify the Heart Allocation System” demonstrated that the policy changes have been successful overall in creating listing statuses that prioritize candidates according to their risk of mortality while awaiting transplant. Specifically, the median time spent waiting prior to transplantation has decreased dramatically, from 242 days pre-policy change to 78 days post-policy change, a 68% decrease [32]. Furthermore, the report found a significant increase in the rate of transplantation, with the most dramatic increase for the most medically urgent candidates. Data also suggest that short- and intermediate-term post-transplant survival is similar to the previous era, with improved survival for patients listed as Status 1.

Given that the new allocation system prioritizes those with temporary MCS over those with uncomplicated durable LVADs, it was expected that there would be an increase in patients BTT from temporary MCS and a decrease in patients BTT from LVADs [13, 27, 28, 33]. However, at the time the new allocation system was implemented, limited data on post-transplant outcomes with various temporary MCS were available to guide decision-making with respect to bridging strategy. Using data from the International Society for Heart and Lung Transplantation Thoracic Registry between 2005 and 2016, Yin and colleagues [13] described an international cohort of 6528 patients who were bridged to HT with the following types of MCS: durable LVADs (n = 6206), VA ECMO (n = 134), temporary pVADs (n = 75), surgically implanted temporary VADs (n = 38) or BiVADs (n = 75). BTT with VA-ECMO or percutaneous temporary VADs was independently associated with higher risk of mortality. The mortality risk was highest after transplant for patients BTT with VA ECMO, with 76% survival within 1-month post-transplant. However, in recipients BTT with VA ECMO who survived past one month after HT, the rate of mortality declined and approximated that of patients BTT with other strategies (76% survival at one month and 71.2% 12 months post-transplant). Importantly, the distribution of the cause of death in patients BTT with VA ECMO was not different from other bridged groups in the study [13].

As expected, the first studies following the implementation of the new allocation system demonstrated that the percentage of patients supported by durable LVAD at listing had significantly declined, while patients BTT with VA-ECMO had significantly increased [31, 34, 35]. In an analysis of the UNOS registry, Jawitz *et al*. [31] found that the percentage of patients BTT with temporary MCS increased from 13.5% to 44.5%, while those BTT from a durable LVAD decreased from 41.8% to 21.2%, despite a 6.8% increase in LVAD implantations from 2018 to 2019. These findings were validated by other national retrospective reviews using the UNOS database, with the increase in pre-transplant temporary MCS driven by the more frequent bridging with VA ECMO and a greater than three-fold increase in bridging with IABP [33, 35, 36, 37, 38]. However, it was unclear whether this trend reflected the new prioritization of patients or a shift in treatment practices in order for programs to provide patients with a higher priority status [31]. A study by Parker and colleagues [36] using SRTR data retrospectively compared initial HT candidates before and after the change to the allocation system, and found transplant centers listed more candidates with VA ECMO, IABPs, and exception requests and fewer candidates with inotrope therapy. The new distribution of statuses was not explained by baseline characteristics alone, suggesting that changes to the allocation system may have undue influence on treatment and support strategies, although further studies will be needed to fully understand this phenomenon.

### 4.2 Bridging with VA ECMO

Over the last decade VA ECMO has been increasingly employed in critically ill patients as a bridge to durable MCS and ultimate HT (bridge-to-bridge strategy) [39, 40, 41, 42, 43]. However, prior to the implementation of the new allocation system, the use of VA ECMO as a BTT was extremely infrequent, as outcomes have been historically poor. An analysis of the UNOS database between 2003 and 2016 revealed that of 25,168 adult HT recipients, only 107 (0.4%) patients were BTT with VA ECMO; by comparison, 6148 (24.4%) patients were BTT with a durable LVAD [44]. BTT with VA ECMO was associated with an increased risk of early- and mid-term mortality, as well as an increased risk of primary graft dysfunction or failure [44]. Other studies of the UNOS database and Extracorporeal Life Support Organization (ELSO) Registry had similar findings: 30-day mortality ranged from 27%–34% and 1-year survival was 53%–58% in this patient population [45, 46]. Even though patients supported with VA ECMO comprise a small portion of patients listed for HT, in recognition of the attendant risks to this bridging strategy, specifically a higher waitlist mortality, under the new allocation system these patients are assigned the highest priority (Status 1).

While waitlist time and mortality both decreased immediately following implementation of the new allocation system, there was an initial reservation that this came at the expense of post-transplant survival [47]. The most critically ill patients with the lowest chance of long-term survival are now the first to be transplanted. Additionally, Status 1 and 2 patients now have access to wider sharing of organs, raising the potential for increased procurement distances and longer graft ischemic times, both of which are associated with decreased survival and an increased risk of primary graft dysfunction [48, 49, 50]. However, concerns for decreased post-transplant survival appear to be unfounded, as 1-year survival has remained comparable to the non-ECMO population in the short time period since the allocation change.

In a UNOS database analysis from November 1, 2015 to September 30, 2019, Loyaga-Rendon *et al*. [51] analyzed 296 patients supported with VA ECMO at the time of listing for HT, 191 (64.5%) listed under the previous allocation system and 105 (35.5%) listed under the new allocation system. Patients listed after October 2018 had a higher cumulative incidence of HT (*p* < 0.001), lower incidence of waitlist mortality or removal due to clinical deterioration (*p* = 0.001), and increased 6-month survival after transplantation, 90.6% vs. 74.6%, respectively (*p* = 0.002) [51]. Hess and colleagues [52] similarly reviewed the UNOS database for patients BTT with VA ECMO over a longer timeframe, between 2010 and 2021. Despite this longer time frame, the number of patients analyzed was similar to the study by Loyaga-Rendon, evidence for the growing use of VA ECMO prior to HT. Of 285 patients, 173 (60.7%) were listed under the old allocation system and 112 (39.3%) were listed under the new allocation system. In this study, those BTT with VA ECMO had decreased length of time on the waitlist and greater likelihood of eventual HT, without a significant difference in one-year post-transplant survival compared to the old allocation system (79.8% vs. 90.3%; *p* = 0.3917). Notably, increasing body mass index (BMI), worsening renal function, and preoperative respiratory failure requiring mechanical ventilation were all recipient-specific risk factors for increased mortality. One of the more recent analyses of the UNOS database by Elde *et al*. [53] compared patients listed and transplanted before and after the implementation of the new allocation system (October 18, 2017–October 17, 2018: 1606 patients vs. October 18, 2018–October 17, 2019: 1841). The authors found that graft ischemic times were longer in the new era, as donor organs came from significantly farther distances. The number of patients BTT with VA ECMO increased almost five-fold since October 2018 (4.0% vs. 1.0%). Despite their increased risk profile, the 180-day post-transplant mortality rate for patients BTT with VA ECMO improved from 28.6% to 8.4% [53].

There are potential confounding factors to consider when reviewing early promising outcomes of patients BTT with VA ECMO following the implementation of the new allocation system. Given the increase of VA ECMO utilization, it may be the case that VA ECMO is now being utilized to bridge a less critically ill cohort of patients as a means of improving their candidacy for HT. Similarly, the new allocation system specifically prioritizes patients on non-dischargeable MCS devices, which has most likely contributed to shorter time spent on the waitlist and a decreased risk for complications associated with prolonged VA ECMO support.

### 4.3 Bridging with Intra-aortic Balloon Pump

Prior to the new allocation system studies on IABP use as a BTT were limited, although they demonstrated safety, improvement in hemodynamics, and comparable post-transplant mortality [54]. The new allocation system increased the prioritization of patients BTT with IABP and strict hemodynamic criteria to Status 2, and unsurprisingly the use of IABP as a BTT increased by more than 300% [55]. The rapid increase in IABP as BTT has been posited to be a reflection of its ease of use and perceived sustainability as a bridging strategy relative to other temporary MCS. IABPs may be inserted percutaneously through the axillary or subclavian arteries, a configuration that allows patients to ambulate and participate in physical rehabilitation while awaiting HT [56, 57]. It has been suggested that the increase in IABP use prior to HT is a result of provider bias, and that the baseline characteristics of these patients, their waitlist mortality, and their post-transplant outcomes would reflect this paradigm shift [38, 58].

One of the first studies to evaluate the impact of the 2018 allocation system on patients BTT with IABP was conducted by Huckaby and colleagues [38]. Patients who underwent HT between 2013 and 2019 were stratified based on temporal relation to the change in allocation, and a total of 1342 (8.6%) of patients were BTT with IABP. The rates of BTT with IABP increased significantly after the policy change (7.0% vs. 24.9%, *p* < 0.001). As expected, after the new allocation system was implemented, patients spent fewer days on the waitlist (15 vs. 35 days, *p* < 0.001), had longer ischemic times (3.5 vs. 3.0 hours, *p* < 0.001), and received organs from a greater distance (301 vs. 105 miles). After October 2018, patients who were bridged with IABP were more likely to survive transplantation (76.4% vs. 89.8%, *p* < 0.001). Among those patients who died on the waitlist, multiorgan failure was significantly less common, suggesting that the use of IABP as a bridging strategy may improve organ perfusion in select patients. Importantly, baseline recipient characteristics for patients BTT with IABP before and after the new allocation system remained similar, allaying concerns about gaming of the system.

These findings were subsequently confirmed in a more focused study by O’Connell and colleagues [55]. The authors reviewed the UNOS database for patients with IABPs listed or transplanted in the two years before and after the new allocation system was implemented (October 18, 2016–October 17, 2018 vs. October 18, 2018–September 4, 2020) [55]. A total of 2358 patients who met inclusion criteria, and patients with IABPs who were listed in the new allocation system era were both more likely to receive HT and spend less time on the waitlist, with no difference in one-year post-transplant survival between eras (*p* = 0.056) despite longer ischemic times and donor travel distances. This study also found that among patients listed with IABP, non-transplanted patients had comparable waitlist mortality but an increased probability of delisting under the new allocation system and were also more likely to be delisted due to clinical decompensation. This suggests that IABP is being used appropriately in critically ill patients listed for HT.

Most recently, Hanff and colleagues [59] observed under the new allocation system, there is significant heterogeneity among patients listed as Status 2 and some subgroups may have waitlist mortality risk more closely approximating Status 1 or Status 3. Moreover, there is little evidence to guide the escalation of therapy in these patients (e.g., continued inotropic support vs. insertion of IABP vs. initiation of other temporary MCS). Patients BTT with IABP are thought to represent a lower-risk group of patients within Status 2. In this study, the UNOS database was retrospectively analyzed for all patients listed as Status 2 under the current allocation system (n = 3638). The authors sought to compare the risk of waitlist death or delisting within Status 2 across each listing criteria and to compare Status 2 subgroups to patients listed as Status 1 or as Status 3 due to high-dose inotropes (Inotrope Status 3). Status 2 patients listed with IABP, durable LVAD malfunction, non-surgical temporary MCS, and status exceptions had comparable rates of waitlist mortality or decompensation as patients listed as Inotrope Status 3. The results highlighted that the decision between temporary MCS (e.g., IABP) and high-dose inotropes may be arbitrary or subjective, and the prioritization of IABP-induced preferential utilization in circumstances where inotropes may be sufficient. It is worth noting that the new heart allocation system provides some protection against this by requiring specific hemodynamic criteria for initiating temporary MCS. Even so, additional studies are needed to understand and appropriately categorize the risk of patients bridged with IABP compared to other patients supported with either temporary MCS or inotrope therapy.

### 4.4 Bridging with Durable LVADs

Durable LVADs have improved survival in patients awaiting HT while enabling recovery of end organs for those patients who would otherwise be ineligible for transplantation. Recent advancements in LVAD technology have led to improved long-term survival that now approaches 85% to 88% at 1 year and 49% to 54% at five, respectively [21]. However, despite having a priority in the previous allocation system, less than half of patients with LVADs implanted as either a BTT or BTC progressed to HT; under the new allocation system, these numbers are further reduced. The modifications made to the allocation system were intended to reflect the relative stability of patients with durable LVADs and to give increased priority on the waitlist to patients on temporary or other non-dischargeable MCS. Since the demotion of durable LVADs to primarily Status 4 (aside from 30 discretionary days at Status 3), the annual number of LVAD implants has steadily declined and the role of LVAD as a BTT strategy has been significantly diminished.

A study by Yarboro *et al*. [60] sought to identify what factors predict progression to successful HT after LVAD implant as a means to inform patient care following the implementation of the new allocation system. All patients in the STS Intermacs (Interagency Registry for Mechanically Assisted Circulatory Support) database between January 1, 2010 and December 31, 2019 and who received a durable LVAD as either a BTT (n = 5242; 45.6%) or BTC (n = 6248; 54.3%) strategy were analyzed. The group was further subdivided into patients who were implanted before (n = 10,588; 92.1%) and after (n = 902; 7.9%) changes to the allocation system took effect. Of 11,490 patients, 45.5% progressed to HT, most within 14 months after LVAD implant. Progression was better for patients with an LVAD who were classified as BTT patients (53.0%) compared to patients with an LVAD classified as BTC patients (36.6%). Under the new allocation system, progression to HT was significantly lower at 14 months (18.6% vs. 34.8%, *p* < 0.001). Being listed for HT at the time of implant was associated with successful progression to transplant both before and after changes to the allocation system. Thus, it is now very unlikely that patients implanted with a durable LVAD as a BTT or BTC strategy will ever progress to HT, barring a serious LVAD complication that warrants higher status prioritization. Under the current allocation system, durable LVADs may now be most appropriate for true DT or in high-risk patients in whom BTC or bridge-to-decision are the only viable options [61].

### 4.5 Bridging with Impella

For patients who require additional support beyond IABP, an additional option for BTT is the Impella device, a transvalvular micro-axial flow pump that improves the hemodynamic profile through mechanical unloading of the left ventricle. Impella is typically inserted through the common femoral or axillary artery via a surgical cut-down and advanced across the aortic valve into the left ventricle. Notably, axillary Impella facilitates ambulation and prehabilitation while patients await either transplantation or durable LVAD. For patients with refractory CS, Impella can provide hemodynamic support and potentially reverse end-organ dysfunction in patients who are waitlisted for HT [62]. Seese and colleagues [63] evaluated all adult recipients in the UNOS registry who were BTT with Impella 5.0 between 2010 and 2018. Of 236 patients who were listed with Impella 5.0 support, 57 (24%) patients were successfully bridged to heart transplant and 87 (37.0%) patients were bridged to durable continuous-flow LVAD, while 47 (20%) patients were removed from the waitlist for death or clinical deterioration. Early and late post-transplant survival was excellent: 96.5% at 30 days, 93.8% at 90-days, and 90.3% at 1-year. Following the policy change, there has been a four-fold increase in the use of the Impella [63].

In the new UNOS allocation era, several studies have demonstrated excellent outcomes among patients directly BTT with Impella. A 2022 study by Pahwa *et al*. [64] queried the UNOS database for patients BTT with Impella before and after the new allocation system and found Impella use as a bridging strategy increased substantially over a four-year time period, from 1% (2015) to 4% (2019) (*p* < 0.01). Moreover, the most substantial increase in the use of Impella occurred after October 2018. Compared to pre-allocation change, patients bridged with Impella post-allocation change had significantly fewer waitlist days (12 days vs. 45 days, *p* < 0.01), were more likely to be directly transplanted (80% vs. 56%, *p* < 0.01), had significantly lower waitlist mortality (13% vs. 25%, *p* < 0.01), and were less likely to be converted to durable LVAD (3% vs. 13%, *p* < 0.01) [64]. The study also reported that post-transplant survival of patients bridged with Impella was not adversely impacted after the policy change, and concluded that Impella use would become a lasting strategy for bridging under the new allocation system. This trend was confirmed in a publication by Cevasco and colleagues [65] examining the newest generation Impella 5.5, which received Food and Drug Administration (FDA) approval in 2019. In this retrospective review of the UNOS database 464 patients were BTT with Impella 5.5, and 378 (81%) patients were directly bridged with the device. Device complications and failure were uncommon, and waitlist death (7%) and clinical deterioration (5%) were the most common reasons for waitlist removal [65]. The most common complication post-transplant was acute kidney injury requiring dialysis (16%), and one-year post-transplant survival was excellent (89.5%).

## 5. Conclusions

Modifications to the U.S. heart allocation system implemented in October 2018 were intended to better prioritize candidates on the waitlist according to medical urgency, reduce clustering of candidates assigned to the top-tier status, decrease waitlist times, and provide more equitable geographic access to donors. Even though the new system has been in effect for only a brief period of time, it has had a tremendous impact on the strategies used to bridge patients with heart failure to eventual transplantation. Future studies should focus on safely bridging patients from VA ECMO, IABP, Impella, and other forms of temporary MCS. Additionally, further adjustments to the allocation system may be needed to more appropriately risk stratify Status 2 patients. As the use of durable LVADs continues to transition from a bridging strategy to a DT therapy, optimizing these devices and the patients in whom they are implanted will be critical for their long-term success.

## Abbreviations

BTC, bridge to candidacy; BTT, bridge to transplant; CS, cardiogenic shock; DT, destination therapy; ELSO, Extracorporeal Life Support Organization; HF, heart failure; HT, heart transplants; INTERMACS, Interagency Registry for Mechanically Assisted Circulatory Support; LVAD, left ventricular assist device; MCS, mechanical circulatory support; OPTN, Organ Procurement and Transplantation Network; SRTR, Scientific Registry of Transplant Recipients; STS, Society of Thoracic Surgeons; UNOS, United Network for Organ Sharing; VA ECMO, venoarterial extracorporeal membrane oxygenation.
